# Effect of a Spiritual Care Program on Treatment Adherence and Sleep Quality in Hemodialysis Patients: A Cluster‐Randomized Clinical Trial

**DOI:** 10.1002/hsr2.71627

**Published:** 2025-12-14

**Authors:** Naser Parizad, Maryam Rahimpour, Vahid Alinejad, Abdullah Khorami Markani

**Affiliations:** ^1^ Patient Safety Research Center Clinical Research Institute Urmia University of Medical Sciences Urmia Iran; ^2^ School of Nursing and Midwifery Urmia University of Medical Sciences Urmia Iran; ^3^ Biostatistics and Epidemiology School of Medicine Urmia University of Medical Sciences Urmia Iran; ^4^ Department of Nursing Faculty of Nursing and Midwifery Khoy University of Medical Sciences Khoy Iran

**Keywords:** cluster‐randomized trial, hemodialysis, patient, sleep quality, spiritual care, treatment adherence

## Abstract

**Background and Aims:**

Declining sleep quality is common among hemodialysis patients, adversely affecting their quality of life and treatment adherence. Improving sleep is a nursing priority, and targeted interventions might be beneficial. This study aimed to evaluate the effect of a spiritual care program on treatment adherence and sleep quality in hemodialysis patients.

**Methods:**

This study was a parallel, two‐group, cluster‐randomized clinical trial with a pretest–posttest design. In 2023, 80 hemodialysis patients from two teaching hospitals in Urmia, Iran, were enrolled via convenience sampling and cluster‐randomized by hospital unit to intervention (*n* = 39) or control (*n* = 37 completers) groups. The intervention involved four 1 h spiritual care sessions twice weekly post‐dialysis. Outcomes were measured using the End‐Stage Renal Disease Adherence Questionnaire (ESRD‐AQ) and Pittsburgh Sleep Quality Index (PSQI) at baseline and 1 month post‐intervention. Data were analyzed in SPSS v26.0. Normality was checked with Kolmogorov−Smirnov, between‐group differences with independent *t*‐tests, and within‐group changes with paired *t*‐tests.

**Results:**

The mean age of the 76 participants was 52.09 years (range: 20–84 years); 65 participants (85.5%) were male, and 11 (14.5%) were female. Baseline scores showed no significant between‐group differences (adherence: *p* = 0.848; sleep quality: *p* = 0.891). Post‐intervention, the intervention group had significantly higher adherence (mean difference: 219.60 [95% CI: 159.51–279.69]; *p* < 0.001; Cohen's *d* = 1.92) and better sleep quality (mean difference: −2.67 [95% CI: −1.46 to −3.88]; *p* = 0.006; Cohen's *d* = 0.68) compared to the control group. Within the intervention group, adherence improved (*p* < 0.001; Cohen's *d* = 2.15) and sleep quality enhanced (*p* < 0.001; Cohen's *d* = 0.79); no changes were observed in the control group (*p* > 0.05).

**Conclusion:**

A spiritual care program improves treatment adherence and sleep quality in hemodialysis patients. Hospital administrators should integrate spiritual interventions like meditation and counseling, considering barriers such as staffing. Future studies should include diverse populations and longer follow‐ups.

**Reporting Tool:**

The CONSORT 2010 checklist was used.

## Summary

- What does this paper contribute to the wider global community?
- The Spiritual Care Program can improve treatment adherence and enhance sleep quality in hemodialysis patients.
- This study's results emphasize the importance of spiritual care for patients receiving hemodialysis.
- This report urges health authorities to take spiritual care seriously for hemodialysis patients.
- As the number of hemodialysis patients increases in Iran and worldwide, these findings could significantly help reduce complications and mortality rates.

## 1 Introduction

End‐stage renal disease (ESRD) is primarily managed through dialysis or kidney transplantation [1]. Hemodialysis is the most commonly used treatment modality for chronic kidney failure [2]. Successful hemodialysis depends on multiple factors, including adherence to dietary restrictions, compliance with prescribed medications, fluid intake limitations, and regular attendance at dialysis sessions [3]. Despite its crucial role in supporting life, hemodialysis is linked to various complications, notably sleep disturbances [4].

Although sleep disturbances are highly prevalent among hemodialysis patients, they are often overlooked [5]. The reported prevalence of sleep disorders in these patients is approximately 80% [6]. Such disturbances can lead to neuropsychiatric issues, chronic fatigue, reduced quality of life, and higher mortality rates [7]. Research has also shown that sleep disorders contribute to depression and anxiety in hemodialysis patients, which in turn can negatively affect adherence to treatment regimens [8, 9].

Nonadherence to treatment regimens is a significant concern among hemodialysis patients [9]. Adherence to prescribed treatment is crucial for achieving optimal therapeutic outcomes, reducing complications—such as nutritional disorders, muscle cramps, and bloodstream infections—and ultimately lowering mortality rates [10]. However, more than half of hemodialysis patients fail to comply with their prescribed dietary and fluid restrictions [3]. In a study by Zolfaghari et al. [11], despite the implementation of a cognitive‐behavioral intervention, only 17.6% of patients adhered to dietary restrictions, and 47.1% complied with fluid intake limitations [11].

Multiple factors influence treatment adherence in hemodialysis patients, including patient‐related factors (e.g., knowledge about dietary restrictions, health beliefs, and attitudes toward treatment), disease‐ and treatment‐related factors, socioeconomic status, psychological factors, and cultural differences [12]. Since hemodialysis patients often have low health literacy, limited control over their condition, and encounter more severe disease complications, the implementation of educational and supportive care programs can help them reduce anxiety, enhance treatment adherence, minimize disease‐related complications, and lower healthcare costs [13].

Beyond physical challenges, hemodialysis patients also face significant emotional and spiritual difficulties [14]. Spiritual well‐being has been shown to positively influence mental health, improve sleep quality, and encourage better adherence to treatment [11]. Consequently, addressing the spiritual needs of hemodialysis patients and fostering their spiritual well‐being has been recommended as an effective strategy to help them cope with disease‐related stress and mitigate various challenges [15].

Spirituality is recognized as a relatively new dimension of health that plays a crucial role in patient recovery alongside physical, mental, and social well‐being [16]. Without spiritual health, the other dimensions of health may not function optimally; consequently, disruptions in spiritual health can lead to psychological distress, depression, and a loss of life meaning. These consequences can ultimately impede the attainment of a high quality of life [17]. Puchalski et al. [18] reported that spiritual health assists individuals in adapting to illness [18], while Balboni et al. [19] emphasized that religious beliefs and spirituality gain heightened importance during periods of illness [19]. Recent studies have highlighted the significant role of spirituality in chronic conditions. Şanli et al. [20] reported that higher levels of spirituality were associated with lower anxiety and slightly greater resilience among hemodialysis patients [20]. Similarly, Yıldırım Üşenmez et al. [21] found a weak positive correlation between spirituality and resilience in patients with breast cancer [21]. Moreover, Korkmaz et al. [22] demonstrated that spirituality exerted a protective effect against death‐related depression, anxiety, and loneliness in young adults [22]. Although spiritual health is relevant to all patients, it is especially significant for those with chronic conditions, including hemodialysis patients at the end stages of life [23]. In fact, the World Health Organization (WHO) considers spiritual health the most important among the four dimensions of human health, underscoring that its achievement necessitates attention to spirituality and spiritual needs [24]. Given that nursing is a holistic discipline addressing all facets of human existence, understanding this dimension of patient care is vital for nurses [25]. Since the overarching goal of nursing involves maintaining and promoting health, preventing disease, and alleviating patient anxiety, spiritual care plays a key role in realizing these objectives [26]. Consequently, addressing the spiritual needs of patients should be a core nursing responsibility, and spiritual care must be regarded as a fundamental nursing intervention [27].

This study is guided by the biopsychosocial‐spiritual model, which conceptualizes health as the dynamic interaction of biological, psychological, social, and spiritual dimensions [28]. This framework proposes that spiritual well‐being—encompassing meaning, purpose, and connection to a higher power—significantly influences coping mechanisms, mental health, and treatment outcomes in chronic illnesses such as ESRD. By addressing spiritual needs, this model suggests that interventions may reduce anxiety, enhance resilience, and improve adherence and sleep quality, particularly among hemodialysis patients experiencing physical and emotional stressors [20, 22].

Considering the high prevalence of both treatment nonadherence [11] and sleep disorders [9] among hemodialysis patients, and in view of the limited research exploring the effect of spiritual care programs on treatment adherence and sleep quality, particularly within the context of Iranian hemodialysis patients, further comprehensive studies are warranted. Therefore, this study was designed to assess the impact of a spiritual care program on the quality of sleep and treatment adherence among patients undergoing hemodialysis. The study hypothesis posited that the implementation of a spiritual care program can improve treatment adherence and sleep quality among hemodialysis patients.

## 2 Methods

### 2.1 Study Design and Setting

This cluster‐randomized, parallel‐group, two‐arm clinical trial with a pretest−posttest design was conducted in 2023 at hemodialysis units of Taleghani and Imam Khomeini teaching hospitals in Urmia, northwestern Iran. Units were randomized to intervention/control using computer‐generated random numbers. After obtaining approval from the Research Deputy and the Review board of Urmia University of Medical Sciences, the research was registered in the Iranian Registry of Clinical Trials (IRCT) (Registration No.: IRCT20131112015390N9).

### 2.2 Sampling and Participants

The sample size calculation was based on the results of a study by Zhianfar et al. [29], which indicated post‐intervention mean adherence scores (±standard deviation) of 531.06 ± 85.93 for the intervention group and 470.45 ± 112.75 for the control group. Using a statistical power of 80% and a confidence interval of 95%, the minimum necessary sample size was determined to be 35 participants per group using the following formula. Considering an anticipated dropout rate of 15%, 40 participants were allocated to each group, resulting in a total sample size of 80 participants.

$$n=\frac{{\left(Z_{1-\frac{\alpha }{2}}+Z_{1-\beta }\right)}^{2}\left(\delta_{1}^{2}+\delta_{2}^{2}\right)}{{\left(\mu_{1}-\mu_{2}\right)}^{2}}$$

$$n=\frac{{\left(1.96+0.84\right)}^{2}(85.93^{2}+112.75^{2})}{{\left(531.06-470.45\right)}^{2}}=35$$

**Figure 1:**
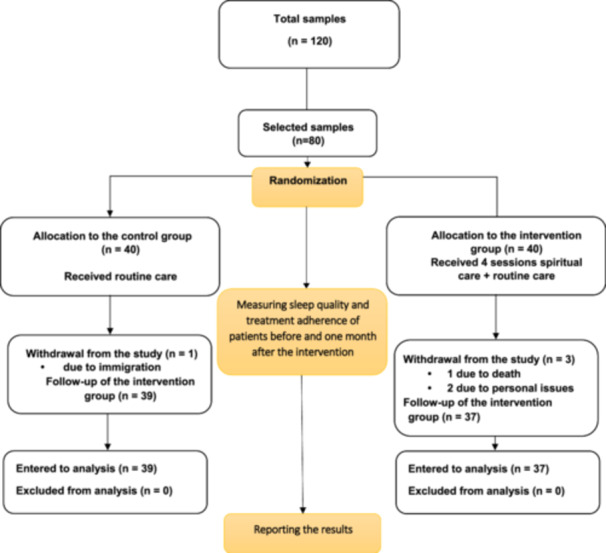
CONSORT flow diagram of a randomized clinical trial of a spiritual care program for hemodialysis patients. This flowchart depicts participant flow in a 2023 randomized, two‐arm, pretest−posttest trial at hemodialysis units in Urmia, Iran, evaluating a spiritual care program's effect on treatment adherence (ESRD‐AQ, 0–1200, higher scores = better adherence) and sleep quality (PSQI, 0–21, lower scores = better sleep). Of 120 assessed patients, 30 were excluded (did not meet criteria), and 10 declined. Eighty patients were randomized (40 per group) to the intervention (four 1 h spiritual care sessions over 2 weeks) or control (routine care). All participants completed the baseline assessments; however, four patients were lost to follow‐up at the 1‐month post‐intervention assessment (three in the intervention group and one in the control group). The remaining participants completed the ESRD‐AQ and PSQI assessments and were included in the analysis in accordance with CONSORT 2010 guidelines.

Of 120 patients, 30 did not meet the inclusion criteria, and 10 refused participation (Figure 1). The inclusion criteria for participants included: (a) a confirmed diagnosis of chronic kidney failure requiring regular hemodialysis as determined by a nephrologist, (b) having an established medical record at the hospital, (c) undergoing hemodialysis three times per week (3–4 h per session), (d) having 32−68 years of age, (e) willingness to participate in the study, (f) ability to hear and speak, (g) literacy in reading and writing, and (h) Muslim religious affiliation.

Exclusion criteria included the following: (a) unwillingness to remain in the study for any reason, (b) clinical deterioration necessitating transfer from the hemodialysis unit or admission to the intensive care unit (ICU) during the study period, (c) patient death, and (d) undergoing any procedures beyond routine treatment during the study period.

The allocation of Taleghani and Imam Khomeini Hospitals to the intervention and control groups was performed using simple randomization via a computer‐generated random number sequence. Allocation concealment was maintained using opaque, sealed envelopes to prevent bias during the randomization process.

### 2.3 Data Collection

Data collection was performed using three questionnaires: a demographic questionnaire, the End‐Stage Renal Disease Adherence Questionnaire (ESRD‐AQ), and the Pittsburgh Sleep Quality Index (PSQI).

#### 2.3.1 Demographic Questionnaire

The demographic questionnaire included information on age, gender, education, residence status, marital status, income level, and occupation. This questionnaire was completed through interviews conducted with the patient and an accompanying caregiver.

#### 2.3.2 ESRD‐AQ

This questionnaire was developed and validated by Kim et al. in the United States of America in 2010. The questionnaire includes 46 items divided into five sub‐scales: (i) General Information (5 items), (ii) Hemodialysis Treatment Adherence (14 items), (iii) Medication Adherence (9 items), (iv) Adherence to Fluid Restrictions (10 items), and (v) Adherence to Dietary Restrictions (8 items). However, not all items contribute to the final scoring; only six items (14, 17, 18, 26, 31, and 46) are used to calculate the adherence score. Participants reply to these items on a five‐point Likert scale scored as follows: “1 = 200”, “2 = 150,” “3 = 100,” “4 = 50,” and “5 = 0.” The total adherence score was obtained by summing these relevant items, with values ranging from 0 to 1200, where higher scores indicated better treatment adherence. Kim et al. [30] reported a content validity index (CVI) of 0.99 and an internal consistency coefficient (Cronbach's *α*) of 0.91 for this instrument [30]. The questionnaire was previously adapted for Iranian hemodialysis patients by Kalili et al. [31], who confirmed its reliability with a Cronbach's *α* of 0.75 [31]. “In this study, a pilot test with 30 participants using a 2‐week test–retest method showed a reliability of 0.89.”

#### 2.3.3 PSQI

It was created by Buysse et al. [32] and contains 19 items assessing seven dimensions of sleep quality: overall sleep quality, duration of sleep, sleep latency, sleep efficiency, sleep disturbance, need meds to sleep, and day dysfunction due to sleepiness. Responses are scored on a 4‐point Likert scale ranging from “0 = *Better Sleep*” to “3 = *Worse Sleep.*” The PSQI total score varies between 0 and 21, where lower scores signify better sleep quality and higher scores indicate worse sleep quality. Buysse et al. [32] reported a sensitivity of 0.89, specificity of 0.69, and an internal consistency coefficient (Cronbach's *α*) of 0.83 for the instrument [32]. The Persian version of the PSQI has also been validated by Torabi et al. [33], who found a Cronbach's *α* of 0.89 [33]. In this study, a pilot test involving 30 participants and employing a 2‐week test–retest procedure demonstrated a reliability of 0.86.

### 2.4 Study Procedure

This study is a parallel, two‐group, clustered‐randomized with a pretest–posttest design. In the initial phase, after obtaining approval from the Ethics Committee of Urmia University of Medical Sciences and receiving the IRCT registration code, the researcher (M.R.) visited Taleghani and Imam Khomeini Hospitals in Urmia with an official introduction letter from the university. Following coordination with the heads of the relevant units and attending physicians, eligible participants were selected using convenience sampling based on predefined inclusion criteria. Specifically, the hemodialysis units at Taleghani and Imam Khomeini Hospitals were randomly allocated to the control and intervention groups.

After sampling, the researcher (M.R.) visited the hemodialysis units, introduced herself to the patients, and explained the study objectives and methodology. All participants provided written informed consent and were assured their information would remain confidential. Therefore, all participants completed baseline assessments by filling in the questionnaires. Routine hemodialysis care continued for participants in both the intervention and control groups.

The spiritual care program was implemented for the intervention group after the researcher (M.R.) established rapport and built trust with patients and their family members by clearly introducing herself and explaining her professional expertise. Such trust was deemed vital to encourage cooperation and active participation in the spiritual care interventions. The program consisted of four 1 h individual sessions conducted twice weekly after the patients' hemodialysis sessions in a designated private room within the hemodialysis unit. The session schedule and contents are summarized in Table 1 [34]. The sessions were structured as follows: Session 1 involved a supportive presence, Session 2 assisted with religious rituals, Session 3 facilitated utilizing support systems, and Session 4 offered a summary and review of previous discussions. Prior to implementing the spiritual care program, an educational care package—including a booklet and a brochure—was developed specifically for hemodialysis patients. This material was created based on an extensive literature review and consultations with a psychiatrist and an expert scholar in religious and spiritual sciences. The educational materials were then distributed to patients in the intervention group. In addition, the researcher (M.R.) collected each patient's or caregiver's landline and mobile phone numbers for follow‐up purposes and to address any questions related to their condition. The control group received no educational or spiritual intervention during the study period. Both groups completed the same questionnaires 1 month after the spiritual care program ended, as the PSQI typically measures intervention effectiveness 1 month following intervention. After completion of the intervention phase and data collection, the educational package was also provided to patients in the control group, accompanied by a scheduled session to answer any questions. The CONSORT 2010 checklist was utilized to guarantee high‐quality reporting in this study [35].

**Table 1:** Spiritual care program for hemodialysis patients.

Sessions	Titles	The content of the session
1st session	Supportive presence	1. Building trust, empathy, and honesty between the nurse and the client to establish proper communication. 2. Listening attentively and carefully to the patient's words, concerns, and anxieties. 3. Providing psychological support to patients. 4. Strengthening people's hope and inner strength. 5. Using positive energy sentences and strengthening healthy and constructive thoughts. 6. Establishing verbal and nonverbal communication with the patient. 7. Answering the patient's questions and explaining the treatment process, providing information and awareness to patients about the disease in order to reduce physical and mental stress. 8. Encouraging patients to use recreational and scenic spaces and perform sports activities according to the treating physician's opinion.
2nd session	Support for religious ceremonies	1. Helping the client find the meaning and significance of life and paying attention to the fact that none of life's events is outside of divine destiny. 2. Providing the necessary facilities for performing religious practices. 3. Encouraging the patient to recite prayers, praying, orison, and the Quran. 4. Encouraging the patient to express their religious beliefs. 5. Encouraging patients to refer to religious clergy.
3rd session	The use of support systems	1. Encourage patients to refer to people with whom they can feel comfortable. 2. Emotional support for patients by companions and medical staff. 3. Advise the patient's companions to help the patient perform their normal, daily responsibilities, such as housework. 4. Encouraging the patient to attend work.
4th session	review and reflection	The educational context from the previous three sessions was summarized, and patients were invited to share their thoughts and questions about the spiritual care they received. If there were any uncertainties or ambiguities about the training provided, efforts were made to clarify these for the patients.

#### 2.4.1 Development and Validation of the Educational Care Package

An educational care package, comprising a 24‐page booklet and a tri‐fold brochure, was developed for Muslim hemodialysis patients to support the spiritual care program, aimed at improving treatment adherence and sleep quality. The content was informed by a literature review [16, 36] and consultations with a psychiatrist, an Islamic scholar, a nephrologist, and two dialysis nurses. Key topics included Islamic spiritual practices (e.g., Salah, Dua, Quran recitation), coping strategies for emotional challenges, and linking spirituality to treatment adherence. Materials were written in Persian at a 6th‐grade reading level, with large fonts and visuals for accessibility.

Validation process:

1. Content validity: Ten experts (including spiritual care researchers, nurses, and a patient advocate) rated the materials for relevance, clarity, simplicity, and cultural appropriateness, yielding a CVI of 0.92 (item‐level: 0.85–1.00, *ϰ* = 0.87). Minor revisions simplified advanced content.
2. Pilot testing: Ten hemodialysis patients tested the materials, providing feedback via interviews (mean satisfaction: 4.5/5). Revisions added patient‐suggested examples and clarified terms.
3. Iterative revisions: Two revision rounds refined content and formatting based on feedback, with test−retest confirming comprehension (> 90%).

The materials were distributed in print (digital optional) and integrated into the four‐session program: Session 1 (hope/supportive presence), Session 2 (religious rituals), Session 3 (support systems), and Session 4 (review/reflection). They reinforced in‐person sessions and supported home‐based learning with follow‐up call access.

#### 2.4.2 Intervention Fidelity

Intervention fidelity was maintained through a pilot‐tested session checklist (Table S1), used by the researcher (M.R.) to ensure protocol adherence; audio‐recording of sessions, with 20% randomly reviewed by a blinded co‐investigator (N.P.) showing high adherence (mean 92%); and a 4 h training led by a certified spiritual care specialist to standardize delivery skills.

### 2.5 Data Analysis

This study employed a single‐blind design, in which the researcher (V.A.) responsible for statistical analysis was blinded to participants' group assignments to minimize bias in outcome assessment. Group allocations (intervention or control) were coded as Group A and Group B in the data set, and the analyst was not informed of which code corresponded to each group until the analysis was complete. Taleghani and Imam Khomeini Hospitals were randomly assigned to the intervention or control group using simple randomization with a computer‐generated random number sequence. Sealed envelopes were used to conceal allocation until baseline assessments were completed, reducing the risk of selection bias. Data analysis was conducted using SPSS software, version 26.0 (IBM Corp., Armonk, NY, USA). The Kolmogorov–Smirnov test confirmed the normality of data distribution. Descriptive statistics, including means and standard deviations, and percentages, were calculated for demographic and outcome variables. The chi‐squared test or Fisher's exact test was performed to compare categorical variables between groups. Paired and independent *t*‐tests were used for within‐group and between‐group comparisons, respectively. To assess the clinical significance of the findings, effect sizes were calculated using Cohen's *d* for the primary outcomes, total treatment adherence, and overall sleep quality. Cohen's *d* was computed for both between‐group (intervention vs. control) and within‐group (pre‐ vs. post‐intervention) comparisons. To address multiple comparisons across the five adherence subscales and seven sleep quality dimensions, a Bonferroni correction was applied, setting the significance threshold at *p* < 0.01 for these analyses. Missing data from dropouts (three in the intervention group and one in the control group) were managed using complete‐case analysis, with sensitivity analyses conducted to evaluate the impact of missing data [37]. For all other statistical tests, a *p* value of less than 0.05 was considered statistically significant.

## 3 Results

Of the 80 participants randomized (40 per group), 76 completed the study (37 intervention, 39 control) due to dropouts (3 intervention, 1 control) (Figure 1). Baseline demographic characteristics showed no statistically significant differences between groups (*p *> 0.05 for all variables), confirming group comparability. The mean age of participants was 52.18 ± 10.20 years (intervention group) and 52.00 ± 10.77 years (control group). Most participants in the two groups were male and married (Table 2). The Kolmogorov–Smirnov test confirmed the normality of all data distributions.

**Table 2:** Demographic characteristics of hemodialysis patients by group.

Variables		Intervention group	Control group	Sig.
		*N* (%)	*N* (%)	
Gender	Female	6 (16.2)	5 (12.8)	a *F* = 0.177, df = 1, *p* = 0.752
	Male	31 (83.8)	34 (87.2)	
Marital status	Single	6 (16.2)	9 (23.1)	*F* = 0.565, df = 2, *p* = 0.781
	Married	30 (81.1)	29 (74.4)	
	Divorced	1 (2.7)	1 (2.6)	
Educational status	High school‐ no diploma	13 (35.1)	14 (35.9)	b *χ* ^2^ = 0.908, df = 2, *p* = 0.667
	High school diploma	20 (54.1)	18 (46.2)	
	College degree	4 (10.8)	7 (17.9)	
Employment status	Housewife	6 (16.2)	5 (12.8)	*χ* ^2^ = 2.816, df = 4, *p* = 0.574
	businessman	10 (27)	6 (15.4)	
	Retired	13 (35.1)	20 (51.3)	
	Employed	6 (16.2)	5 (12.8)	
	Unemployed	2 (5.4)	3 (7.7)	
Monthly income	Insufficient	15 (40.5)	12 (30.8)	*χ* ^2^ = 1.088, df = 2, *p* = 0.617
	Somewhat sufficient	9 (24.3)	9 (23.1)	
	Sufficient	13 (35.1)	18 (46.2)	
**Variables**		**Mean ± SD**	**Mean ± SD**	**Test of sig.**
Age (years)		52.18 ± 10.20	52.00 ± 10.77	c *t* = 0.078, df = 74, *p* = 0.938

a Fisher's exact test.

b Chi‐squared test.

c Independent samples *t*‐test.

### 3.1 Treatment Adherence

There was no statistically significant difference in mean scores between the intervention and control groups prior to the intervention (*p *= 0.85). Post‐intervention, a significant improvement was observed in the intervention group compared to the control group (*p *< 0.001), with a large effect size (Cohen's *d* = 1.65). Within the intervention group, a paired *t*‐test showed a significant increase in mean adherence scores from pre‐ to post‐intervention (+219.6; 95% CI: 164.9–274.3; *p* < 0.001), reflecting a very large effect (Cohen's *d* = 2.15), whereas the control group showed minimal, nonsignificant change (+12.2; 95% CI: −2.83 to 27.19; *p *= 0.11). The overall between‐group mean change was +203.1 (95% CI: 145.5–260.8; *p* < 0.001), indicating a substantial effect of the intervention (Table 3).

**Table 3:** Pre‐ and post‐intervention comparison of treatment adherence and subscale scores between intervention and control groups in hemodialysis patients.

Variable	Groups	Before the intervention	After the intervention	Mean difference	Paired *t*‐test
		Mean ± SD	Mean ± SD	(95% CI)	
Hemodialysis treatment adherence	Intervention	305.40 ± 78.20	389.86 ± 93.06	84.46	*t* = − 5.969, *p* < 0.001, *d* = 1.38
				(55.76, 113.16)	
	Control	299.35 ± 85.35	303.84 ± 79.58	4.49	*t* = − 0.738, *p* = 0.465, *d* = 0.05
				(−7.76, 16.74)	
Independent *t*‐test		*t* = 0.321, *p* = 0.75, *d* = 0.07	*t* = 4.338, *p* < 0.001, *d* = 0.98		
Medication adherence	Intervention	114.86 ± 23.16	162.16 ± 46.25	47.30	*t* = −6.315, *p* < 0.001, *d* = 1.28
				(31.81, 62.79)	
	Control	125.64 ± 34.16	130.76 ± 39.09	5.12	*t* = −1.275, *p* = 0.210, *d* = 0.14
				(−3.03, 13.27)	
Independent *t*‐test		*t* = −1.601, *p* = 0.11, *d* = 0.37	*t* = 3.201, *p* = 0.002, *d* = 0.73		
Adherence to fluid restrictions	Intervention	106.75 ± 17.32	163.51 ± 48.08	56.76	*t* = −7.287, *p* < 0.001, *d* = 1.55
				(39.06, 74.46)	
	Control	112.82 ± 22.11	114.10 ± 22.79	1.28	*t* = − 1.000, *p* = 0.324, *d* = 0.06
				(−1.36, 3.92)	
Independent *t*‐test		*t* = −1.326, *p* = 0.19, *d* = 0.31	*t* = 5.771, *p* < 0.001, *d* = 1.34		
Adherence to diet restrictions	Intervention	151.35 ± 30.01	182.43 ± 29.38	31.08	*t* = −5.551, *p* < 0.001, *d* = 1.04
				(20.31, 41.85)	
	Control	144.87 ± 35.89	146.15 ± 35.13	1.28	*t* = −1.000, *p* = 0.324, *d* = 0.04
				(−1.36, 3.92)	
Independent *t*‐test		*t* = 0.851, *p* = 0.40, *d* = 0.19	*t* = 4.869, *p* < 0.001, *d* = 1.10		
Adherence to treatment	Intervention	678.37 ± 85.62	897.97 ± 139.98	219.6	*t* = −10.229, *p* < 0.001, *d* = 2.15
				(164.90, 274.30)	
	Control	682.69 ± 107.62	694.87 ± 104.21	12.2	*t* = −1.659, *p* = 0.11, *d* = 0.12
				(−2.83, 27.19)	
Independent *t*‐test		*t* = − 0.193, *p* = 0.85, *d* = 0.04	*t* = 7.199, *p* < 0.001, *d* = 1.65		

### 3.2 Sleep Quality

No significant difference was observed between groups before the intervention (*p *= 0.848). After the intervention, a significant difference emerged (*p *< 0.001), with a medium effect size (Cohen's *d* = 0.65), indicating a moderate improvement in the intervention group compared to the control group. Within the intervention group, sleep quality scores improved significantly (−2.67; 95% CI: −4.32 to −1.02; *p* < 0.001, Cohen's *d* = 0.69), while the control group showed no significant change (−0.18; 95% CI: −0.43 to 0.07; *p* = 0.15). The overall between‐group mean change was −2.36 (95% CI: −4.03 to −0.69; *p* < 0.001; Cohen's *d* = 0.65) (Table 4).

**Table 4:** Pre‐ and post‐intervention comparison of sleep quality and subscale scores between intervention and control groups in hemodialysis patients.

Variable	Groups	Before the intervention	After the intervention	Mean difference	Paired *t*‐test
		Mean ± SD	Mean ± SD	(95% CI)	
Overall sleep quality	Intervention	1.54 ± 0.96	1.00 ± 0.96	−0.54	*t* = 3.151, *p* < 0.001, *d* = 0.56
				(−0.89, −0.19)	
	Control	1.74 ± 0.96	1.66 ± 0.95	−0.08	*t* = 1.356, *p* = 0.18, *d* = 0.08
				(−0.20, 0.04)	
Independent *t*‐test		*t* = −0.919, *p* = 0. 36, *d* = 0.21	*t* = −3.107, *p* = 0.003, *d* = 0.70		
Sleep latency	Intervention	1.27 ± 0.83	0.81 ± 0.73	−0.46	*t* = 3.482, *p* < 0.001, *d* = 0.59
				(−0.74, −0.18)	
	Control	1.33 ± 1.03	1.30 ± 1.00	−0.03	*t* = 1.00, *p* = 0.32, *d* = 0.03
				(−0.09, 0.03)	
Independent *t*‐test		*t* = 0.291, *p* = 0.77, *d* = 0.07	*t* = 2.446, *p* = 0.02, *d* = 0.55		
Duration of sleep	Intervention	1.40 ± 0.86	1.16 ± 0.86	−0.24	*t* = 2.165, *p* = 0.04, *d* = 0.28
				(−0.47, −0.01)	
	Control	1.35 ± 1.08	1.30 ± 1.05	−0.05	*t* = 0.628, *p* = 0.53, *d* = 0.05
				(−0.22, 0.12)	
Independent *t*‐test		*t* = 0.205, *p* = 0.838	*t* = 0.655, *p* = 0.514		
Sleep efficiency	Intervention	1.24 ± 0.86	0.83 ± 0.72	−0.41	*t* = 3.402, *p* = 0.002, *d* = 0.52
				(−0.66, −0.16)	
	Control	1.20 ± 0.89	1.25 ± 0.84	0.05	*t* = −1.433, *p* = 0.16, *d* = 0.06
				(−0.02, 0.12)	
Independent *t*‐test		t = 0.189, *p* = 0.85, *d* = 0.04	*t* = 2.302, *p* = 0.02, *d* = 0.52		
Sleep disturbances	Intervention	1.59 ± 0.89	1.10 ± 0.96	−0.49	*t* = 3.176, *p* = 0.003, *d* = 0.53
				(−0.81, −0.17)	
	Control	1.46 ± 0.91	1.38 ± 0.90	−0.08	*t* = 1.780, *p* = 0.08, *d* = 0.09
				(−0.17, 0.01)	
Independent *t*‐test		*t* = 0.641, *p* = 0.52, *d* = 0.14	*t* = −1.288, *p* = 0.20, *d* = 0.29		
Need meds to sleep	Intervention	1.62 ± 0.89	1.40 ± 0.98	−0.22	*t* = 1.753, *p* = 0.09, *d* = 0.24
				(−0.47, 0.03)	
	Control	1.41 ± 1.06	1.43 ± 1.04	0.02	*t* = −1.00, *p* = 0.32, *d* = 0.02
				(−0.02, 0.06)	
Independent *t*‐test		*t* = 0.933, *p* = 0.35, *d* = 0.21	*t* = 0.136, *p* = 0.89, *d* = 0.03		
Day dysfunction due to sleepiness	Intervention	1.35 ± 0.88	1.02 ± 0.86	−0.33	*t* = 2.411, *p* = 0.02, *d* = 0.33
				(−0.61, −0.05)	
	Control	1.38 ± 0.87	1.35 ± 0.77	−0.03	*t* = 0.374, *p* = 0.71, *d* = 0.04
				(−0.20, 0.14)	
Independent *t*‐test		*t* = 0.164, *p* = 0.87, *d* = 0.04	*t* = −1.761, *p* = 0.08, *d* = 0.40		
Total sleep quality	Intervention	10.02 ± 3.86	7.35 ± 3.11	−2.67	*t* = 6.250, *p* < 0.001, *d* = 0.69
				(−4.32, −1.02)	
	Control	9.89 ± 4.29	9.71 ± 4.03	−0.18	*t* = 1.482, *p* = 0.15, *d* = 0.04
				(−0.43, 0.07)	
Independent *t*‐test		*t* = −0.193, *p* = 0.848, *d* = 0.03	*t* = 7.199, *p* < 0.001, *d* = 0.65		

## 4 Discussion

Given the importance of treatment adherence and sleep quality in hemodialysis patients and the risks associated with poor adherence and sleep disturbances, enhancing adherence and sleep quality has consistently been a key nursing objective. Consequently, nursing interventions in these areas are crucial [7, 38]. The present study was conducted to determine the impact of a spiritual care program on treatment adherence and sleep quality among hemodialysis patients. The findings demonstrated that implementing a spiritual care program improved patients' adherence to treatment. The large effect size observed for treatment adherence underscores the substantial clinical impact of the spiritual care program, suggesting it could be a valuable addition to standard care for hemodialysis patients to enhance adherence to treatment regimens. Similarly, the medium effect size for sleep quality indicates a clinically relevant improvement, supporting the integration of spiritual care to address sleep disturbances in this population. These effect sizes highlight the practical significance of the intervention beyond statistical significance, reinforcing its potential to improve patient outcomes.

The present study's findings align with those of Moradi et al. [39], who found that integrative cognitive‐spiritual counseling improved dietary adherence in hemodialysis patients [39]. Saedi et al. [40] reported that effective interventions targeting spiritual well‐being could enhance treatment adherence in patients undergoing hemodialysis [40], which aligns with our observations. Spiritual care fosters hope and reduces anxiety, both of which are known to positively influence adherence to therapeutic regimens [34]. In the same line with this notion, the results of a systematic review by Bayan et al. [41] indicated that therapeutic and spiritual counseling programs increase hope among hemodialysis patients and subsequently lead to improved treatment adherence among this population [41]. The current study's findings are consistent with previous research, which reported a positive impact of spiritual interventions on patient adherence [38, 42]. Furthermore, Musavi Ghahfarokhi et al. [43] identified a correlation between spirituality and hope, which supports improved psychological adjustment to illness [43].

Spiritual well‐being plays an essential role in coping with illness‐related stressors and is associated with a positive outlook, inner balance, a sense of purpose, and hope [44]. Collectively, these factors contribute to improved adaptation to disease and better adherence to treatment [40]. Aggarwal et al. [45] found that participation in prayer and spiritual activities reduced psychological distress in hemodialysis patients [45]. Similarly, Siqueira et al. [46] observed that stronger religious beliefs were associated with higher levels of happiness among hemodialysis patients [46]. These findings suggest that spiritual practices contribute to improved psychological well‐being and coping among hemodialysis patients, consistent with prior studies [34, 47]. The findings suggest that improved treatment adherence can be attributed to the spiritual counseling and intervention program, which emphasized faith in divine compassion, reliance on spiritual support during challenging times, and the perception of life extending beyond physical and material limitations. These components reduced negative emotional states and enhanced participants' adherence to medical recommendations.

Although many studies indicate a positive connection between religiosity, spirituality, and treatment adherence among hemodialysis patients, Berman et al. [48] reported no significant link between religious beliefs and treatment adherence in their US study [48]. One possible reason for this discrepancy is the limited number of non‐religious patients in their sample, as only five participants reported having no religious affiliation. In another study conducted to examine the impact of spirituality on recovery after spinal surgery, researchers concluded that the factors influencing recovery were more strongly linked to patient selection and the methods used in surgery rather than to spiritual factors [49]. Other studies have likewise highlighted insufficient evidence linking spirituality or religiosity directly to patients' overall health and well‐being [50, 51]. A potential explanation for these mixed findings is the reliance on quantitative measures to assess religious and spiritual experiences, which might not entirely reflect the subjective meaning of those experiences. Conducting qualitative studies to explore the spiritual beliefs and lived experiences of hemodialysis patients could therefore provide deeper insights. Moreover, many patients may turn to spirituality as a coping mechanism in response to their illness—a phenomenon that has received relatively little attention in current research and warrants further exploration.

The present study also demonstrated that spiritual care has a positive effect on sleep quality in hemodialysis patients. In line with these findings, Eslami et al. [52] reported a significant positive association between spiritual well‐being and sleep quality in this patient group [52]. Li et al. [53] similarly identified a negative correlation between spiritual well‐being and sleep disturbances. In other words, they indicated that a higher level of spirituality is associated with fewer sleep problems [53]. Spiritual care can enhance psychological well‐being by addressing the spiritual needs of hemodialysis patients and providing them with emotional support and comfort. This, in turn, helps them manage emotional and psychological challenges, such as fear, anxiety, depression, and grief, associated with their illness. Additionally, spiritual care may positively influence physical health and overall well‐being, which could explain its role in reducing sleep disturbances and alleviating insomnia [54]. A review of the literature indicates that individuals with higher levels of spirituality and religiosity often experience better physical health [50, 55]. Patients who engage in religious practices and maintain spiritual beliefs may be better equipped to cope with physical and emotional distress, and high levels of adaptation to distress can ultimately lead to improved sleep quality [56]. Moreover, Eslami et al. [52] found that patients who place a strong emphasis on their spiritual well‐being tend to experience lower levels of depression and anxiety. This emotional improvement contributes to greater peace and comfort, thus enhancing sleep quality [52].

Despite extensive evidence supporting the relationship between spiritual well‐being and sleep quality in patients, Yang et al. [56] found no significant association between religious/spiritual activities and sleep quality in their study [56]. This finding suggests that the connection between the sleep quality and religious or spiritual beliefs among patients undergoing hemodialysis may be both complex and potentially reciprocal. Patients experiencing sleep disturbances may be more inclined than those without sleep issues to seek spiritual support as a coping mechanism, whereas strong spiritual or religious beliefs could contribute to heightened sleep disturbances in certain cases. Previous research indicates that individuals with particularly robust spiritual beliefs sometimes report poorer clinical outcomes compared to those with less intense beliefs [57].

In general, poor treatment adherence and sleep disturbances remain significant challenges for patients with chronic conditions, especially those undergoing hemodialysis. Even existing educational interventions and nursing models have not been able to fully resolve these issues. One overlooked component may be the spiritual dimension of care. Spiritual instability, stemming from inadequate spiritual well‐being, is frequently identified as an underlying factor in various illnesses. Consequently, integrating spiritual care into nursing practice may serve as a vital step in improving treatment adherence and sleep quality. The recognized positive impact of spiritual care on patient recovery has led the American Nurses Association to incorporate spiritual care into its standards and ethical codes and subsequently include it in nursing curricula. This shift is grounded in evidence demonstrating that spiritual care substantially enhances the quality of life for patients with chronic illnesses, including those on hemodialysis [36].

### 4.1 Study Limitations

This study has several limitations. First, the use of convenience sampling may have introduced selection bias, potentially limiting the generalizability of findings to other hemodialysis populations. Second, the inclusion of only Muslim participants restricts the applicability of the results to patients from other religious or cultural backgrounds. Because the spiritual care program was designed to align with Islamic beliefs and practices, the observed improvements in treatment adherence and sleep quality may not directly apply to individuals of different faiths or secular orientations. Moreover, spiritual needs can vary even among those sharing the same religion; thus, future interventions should be adapted to participants' individual levels of religiosity and personal preferences to enhance cultural sensitivity and inclusivity. Third, the single‐blind design, in which participants and intervention providers were not blinded, may have introduced performance bias. Fourth, randomization at the cluster (hospital) level rather than the individual level could have introduced intracluster similarities, potentially affecting internal validity. Fifth, the lack of long‐term follow‐up limits conclusions about the sustainability of the intervention's effects. Sixth, the relatively small sample size may have reduced statistical power. Finally, although a Bonferroni correction was applied to control for multiple comparisons, this conservative approach may have increased the risk of Type II errors, possibly underestimating the intervention's true effects. Future studies should include larger and more diverse samples, employ individual‐level randomization, and incorporate longer‐term follow‐up to enhance the generalizability and robustness of findings.

### 4.2 Model Strengths and Limitations

The biopsychosocial‐spiritual model provides a robust framework for understanding the holistic needs of hemodialysis patients, integrating spiritual well‐being with biological, psychological, and social factors [28]. Its strength lies in its comprehensive approach, which supports tailored interventions like spiritual care to enhance coping, reduce anxiety, and improve treatment adherence and sleep quality, as evidenced by our findings. However, the model's reliance on subjective spiritual assessments can be challenging to quantify, potentially limiting its applicability in diverse populations with varying spiritual beliefs. Additionally, the model may require extensive training for healthcare providers to effectively implement spiritual interventions, which could pose logistical challenges in resource‐constrained settings.

## 5 Conclusion

Nonadherence to treatment regimens and sleep disturbances are common issues among hemodialysis patients, often contributing to disease progression, increased hospitalization rates, and suboptimal dialysis outcomes. Based on the observed positive impact of spiritual care on treatment adherence and sleep quality, regularly scheduled spiritual care programs and activities that foster inner peace among these patients may be particularly beneficial.

## 6 Relevance to Clinical Practice

Health policymakers and hospital administrators are encouraged to develop and implement practical strategies to enhance the spiritual well‐being of ESRD patients undergoing hemodialysis. These interventions can enhance treatment adherence and sleep quality for hemodialysis patients, ultimately improving their overall quality of life. Some recommended interventions include meditation, yoga, and spiritual counseling to cultivate hope, involvement in charitable activities, engagement in personally meaningful hobbies, and structured opportunities for exposure to nature. Implementation may face barriers such as limited time, staffing, and training; these can be addressed through brief interventions integrated into routine care, staff training, group sessions, or collaboration with volunteers and spiritual care providers to ensure feasibility and sustainability.

## Author Contributions

N.P. has contributed to conceptualization, methodology, validation, formal analysis, investigation, writing the original draft, project administration, final approval, and agreement. M.R. has contributed to validation, investigation, writing, review, editing, and final approval and agreement. V.A. has contributed to validation, investigation, writing, review, editing, and final approval and agreement. A.K.M. has contributed to conceptualization, methodology, validation, formal analysis, investigation, writing, review and editing, project administration, and final approval and agreement. All authors have read and approved the final version of the manuscript.

## Ethics Statement

This research was conducted in accordance with the guidelines of the Declaration of Helsinki. Approval was obtained from the Research Review Board of Urmia University of Medical Sciences (Date: 30/08/2023/No: IR.UMSU.REC.1402.175). All the participants signed written informed consent. In case any individuals are named in the Acknowledgments, permission was obtained from all of them, in accordance with ICMJE recommendations.

## Conflicts of Interest

The authors declare no conflicts of interest.

## Transparency Statement

The lead author, Maryam Rahimpour, affirms that this manuscript is an honest, accurate, and transparent account of the study being reported; that no important aspects of the study have been omitted; and that any discrepancies from the study as planned (and, if relevant, registered) have been explained.

## Supporting information


**Supplementary Table 1:** Spiritual care program implementation checklist.

## Data Availability

The data that support the findings of this study are available from the corresponding author upon reasonable request via mariamr1382@gmail.com. Maryam Rahimpour had full access to all of the data in this study and takes complete responsibility for the integrity of the data and the accuracy of the data analysis.
