# Supplementary material for: Effect of a Spiritual Care Program on Treatment Adherence and Sleep Quality in Hemodialysis Patients: A Cluster‐Randomized Clinical Trial
Source: Health Sci Rep. 2025 Dec 14;8(12):e71627. doi: 10.1002/hsr2.71627 (PMC12703117; doi:10.1002/hsr2.71627)
Supplement: Supplementary file 1 — Supplementary Table 1: Spiritual care program implementation checklist. [file HSR2-8-e71627-s001.docx]

# Supplementary Table 1. Spiritual Care Program Implementation Checklist

This checklist ensures standardized delivery of the spiritual care program for hemodialysis patients.

| Session | Element | Details | Completion Check (✓/Notes) |
| --- | --- | --- | --- |
| Session 1:  Supportive Presence | Duration | 60 min (10-min intro, 40-min activities, 10-min wrap-up) |  |
|  | Core Topics | • Build trust and empathy. • Listen to patient concerns. • Provide psychological support. • Foster hope and positive thoughts. • Explain treatment to reduce stress. • Encourage recreational activities. |  |
|  | Engagement Prompts | • What’s on your mind about treatment? • What gives you hope? • How can I support you today? • Any hemodialysis questions? |  |
| Session 2:  Religious Ceremonies | Duration | 60 min (10-min review, 40-min activities, 10-min wrap-up) |  |
|  | Core Topics | • Explore life’s meaning and divine destiny. • Support religious practices. • Encourage prayers/Quran recitation. • Discuss beliefs or clergy referral. |  |
|  | Engagement Prompts | • How does faith help you cope? • Want to practice a comforting ritual? • Interested in religious routines? • Need a spiritual leader connection? |  |
| Session 3:  Support Systems | Duration | 60 min (10-min review, 40-min activities, 10-min wrap-up) |  |
|  | Core Topics | • Connect with supportive people. • Offer emotional support. • Advise on daily tasks. • Encourage meaningful activities. |  |
|  | Engagement Prompts | • Who supports you most? • How can caregivers help daily? • What activities give you purpose? • Ideas to engage your support network? |  |
| Session 4:  Conclusion | Duration | 60 min (20-min review, 30-min discussion, 10-min wrap-up) |  |
|  | Core Topics | • Summarize prior sessions. • Address patient questions. • Clarify uncertainties. • Provide educational materials. |  |
|  | Engagement Prompts | • What stood out from past sessions? • Any spiritual care questions? • How will you apply these ideas? • What was most helpful? |  |
